# Integrating User Preferences for Asthma Tools and Clinical Guidelines Into Primary Care Electronic Medical Records: Mixed Methods Study

**DOI:** 10.2196/42767

**Published:** 2023-02-21

**Authors:** Max Moloney, Madison MacKinnon, Emma Bullock, Alison Morra, David Barber, Samir Gupta, John A Queenan, Geneviève C Digby, Teresa To, M Diane Lougheed

**Affiliations:** 1 Asthma Research Unit Kingston Health Sciences Centre Kingston, ON Canada; 2 Division of Respirology Department of Medicine Queen's University Kingston, ON Canada; 3 Dalla Lana School of Public Health University of Toronto Toronto, ON Canada; 4 Canadian Primary Care Sentinel Surveillance Network Kingston, ON Canada; 5 Department of Family Medicine Queen's University Kingston, ON Canada; 6 Division of Respirology Department of Medicine St Michael's Hospital Toronto, ON Canada; 7 Department of Medicine University of Toronto Toronto, ON Canada; 8 Child Health Evaluative Science The Hospital for Sick Children Toronto, ON Canada

**Keywords:** asthma, electronic medical records, quality improvement, knowledge translation, qualitative analysis

## Abstract

**Background:**

Asthma is a chronic respiratory disease that poses a substantial burden on individuals and the health care system. Despite published national guidelines for the diagnosis and management of asthma, considerable care gaps exist. Suboptimal adherence to asthma diagnosis and management guidelines contributes to poor patient outcomes. The integration of electronic tools (eTools) into electronic medical records (EMRs) represents a knowledge translation opportunity to support best practices.

**Objective:**

The purpose of this study was to determine how best to integrate evidence-based asthma eTools into primary care EMRs across Ontario and Canada to improve adherence to guidelines as well as measure and monitor performance.

**Methods:**

In total, 2 focus groups comprising physicians and allied health professionals who were considered experts in primary care, asthma, and EMRs were convened. One focus group also included a patient participant. Focus groups used a semistructured discussion-based format to consider the optimal methods for integrating asthma eTools into EMRs. Discussions were held on the web via Microsoft Teams (Microsoft Corp). The first focus group discussed integrating asthma indicators into EMRs using eTools, and participants completed a questionnaire evaluating the clarity, relevance, and feasibility of collecting asthma performance indicator data at the point of care. The second focus group addressed how to incorporate eTools for asthma into a primary care setting and included a questionnaire evaluating the perceived utility of various eTools. Focus group discussions were recorded and analyzed using thematic qualitative analysis. The responses to focus group questionnaires were assessed using descriptive quantitative analysis.

**Results:**

Qualitative analysis of the 2 focus group discussions revealed 7 key themes: designing outcome-oriented tools, gaining stakeholder trust, facilitating open lines of communication, prioritizing the end user, striving for efficiency, ensuring adaptability, and developing within existing workflows. In addition, 24 asthma indicators were rated according to clarity, relevance, feasibility, and overall usefulness. In total, 5 asthma performance indicators were identified as the most relevant. These included smoking cessation support, monitoring using objective measures, the number of emergency department visits and hospitalizations, assessment of asthma control, and presence of an asthma action plan. The eTool questionnaire responses revealed that the Asthma Action Plan Wizard and Electronic Asthma Quality of Life Questionnaire were perceived to be the most useful in primary care.

**Conclusions:**

Primary care physicians, allied health professionals, and patients consider that eTools for asthma care present a unique opportunity to improve adherence to best-practice guidelines in primary care and collect performance indicators. The strategies and themes identified in this study can be leveraged to overcome barriers associated with asthma eTool integration into primary care EMRs. The most beneficial indicators and eTools, along with the key themes identified, will guide future asthma eTool implementation.

## Introduction

### Background

Worldwide, the number of individuals diagnosed with asthma is >340 million and has continually increased over a 10-year period [1]. Asthma is diagnosed based on a combination of patient history, physical examination, and objective tests. Asthma poses a substantial burden on individuals and the health care system at large. As the prevalence of asthma increases, the burden of asthma on health care systems worldwide will also increase given that individuals with asthma use considerably greater health care resources than those without asthma, have a poorer quality of life, and have an increased likelihood of having mental illness [2-4].

The major contributors to the burden of asthma on individuals and the health care system are the gaps that exist between published guidelines for asthma diagnosis and actual strategies for diagnosis used in primary care [5]. Although standards for asthma diagnosis are well established, less than half of individuals diagnosed with asthma have a confirmed diagnosis through the use of objective measurements of pulmonary function within 1 year before or 2 and a half years following their original diagnosis [6]. Many differential diagnoses are possible for the symptoms of asthma, creating challenges for clinicians in differentiating between asthma and other respiratory conditions [7]. These issues are compounded by a limited number of validated knowledge translation (KT) strategies along with a limited access to spirometry to effectively support practitioners in the diagnosis (and surveillance) of patients with asthma [8]. The limited availability of pulmonary function tests has also been identified in numerous studies as a major barrier to the accurate diagnosis of asthma [6,9].

The integration of valid and reliable approaches to the diagnosis and surveillance of asthma into electronic medical records (EMRs) using electronic tools (eTools) offers an opportunity to use technology to drive KT. eTools are a form of leveraging technological innovations to support KT. The term eTools encompasses a variety of electronic KT applications, including EMR-integrated eTools, eTools for health care providers, eTools for patients, and mobile apps that can be embedded within or linked to EMRs. The potential benefits of using eTools within EMRs include improved quality of care, outcome monitoring, and performance measurement [10,11]. eTools are computer, mobile, and web-based applications designed to make tasks easier. eTools have become a priority for stakeholders with an interest in improving asthma diagnosis and care as they present a unique opportunity to integrate best-practice clinical guidelines, particularly in primary care [12,13]. Despite these findings, eTools that support health care providers are not widely available. eTools offer an opportunity to improve physician performance and support quality improvement in asthma care [14].

There are many stakeholders involved in the development of new eTools for asthma. Integrating a new asthma tool into EMRs requires collaboration across the entire continuum of care, including the researchers and clinicians developing the tools, practitioners implementing the tool in their practice, patients using the technology, and vendors that facilitate the incorporation of the tool into EMRs.

### Objectives

The purpose of this study was to assess patient and health care provider perspectives on how to integrate clinical guidelines into primary care EMRs using eTools. This study assessed the perspectives of patients, health care providers, and individuals with an understanding of EMR vendors to identify strategies to overcome barriers to integrating asthma tools into EMRs. Using a mixed methods approach, this study outlines the important factors for integrating asthma tools and clinical guidelines into primary care EMRs.

## Methods

### Overview

In total, 2 focus groups were convened for the purposes of this research. The number of focus groups used in the study was predetermined by members of the research team. The first focus group involved 7 participants. In total, 86% (6/7) of the attendees were family physicians, and 14% (1/7) were nurses. The second focus group hosted 6 attendees, including 3 (50%) family physicians, 1 (17%) nurse practitioner, 1 (17%) registered nurse, and 1 (17%) patient attendee. Some participants in the focus groups were familiar with each other; however, most participants did not know the other attendees. Participants were recruited from the OntarioMD Peer Leader Program, a subsidiary of the Ontario Medical Association that provides a network of physicians and allied health professionals who are expert users of EMRs and eTools [15]. Potential participants were contacted via email by OntarioMD. Participants included experts from a wide range of family medicine practices across Ontario, including both urban and rural centers as well as small and large practices. The focus groups were held in a web-based format using Microsoft Teams (Microsoft Corp). In total, 2 surveys were designed by the research team, and 1 survey was administered during each focus group.

### Study Design

#### Focus Groups

Data were captured via two 2-hour focus groups using Microsoft Teams. The focus groups followed a directed discussion format, which provided opportunities for the participants to share their experiences and opinions on a set of questions developed by the research team. The first focus group centered on how to integrate an asthma surveillance system, asthma indicators, and clinical guidelines into primary care EMRs. The second focus group aimed to evaluate the asthma eTools developed by the Asthma Research Unit (ARU) to understand the benefits and limitations of various eTools. The focus groups were led by a skilled moderator who encouraged participants to discuss and share their thoughts on topics and questions pertaining to the research question. The discussion was divided into 3 sections, each with a unique goal that contained questions designed to facilitate responses to answer the research question: how to best integrate asthma tools and clinical guidelines into primary care EMRs. The second focus group included a presentation of tools developed by the ARU to demonstrate current eTools for asthma care. The discussions during the focus groups were facilitated by a third-party professional from OntarioMD. The moderator moved the discussion forward when participants had no more to say on the question posed. The workshops were recorded, and a note taker was present throughout.

#### Surveys

Participants completed 2 surveys in addition to taking part in the focus groups. The surveys were administered and collected anonymously. The first focus group dedicated a portion of the time to evaluating various asthma indicators used to assess adherence to best-practice guidelines via the Asthma Indicator Survey. This survey was completed by all 7 participants during the first focus group. The Asthma Indicator Survey solicited ratings of 1 to 5 on a Likert scale from participants on the clarity, relevance, feasibility, and overall perception of potential indicators of asthma in primary care. The survey provided indicators from 2 sources, the Primary Care Asthma Performance Indicators (PC-API) and the Health Quality Ontario (HQO) Asthma Measurement Guide [11,16]. In total, 9 indicators overlapped between the PC-API and HQO indicator lists. For instances in which the PC-API and HQO had similar indicators, participants were asked to select which indicator and definition they preferred. Specific outcomes worthy of consideration could be selected from the Asthma Indicator Survey based on physicians’ views on the indicators best suited to evaluate outcomes for asthma in primary care.

A second survey recorded participants’ evaluations of various eTools for asthma that were demonstrated during the second focus group. The eTools evaluated in the survey included the Asthma Action Plan Wizard, Asthma Management and Outcomes Monitoring System, AsthmaLife portal, Electronic Asthma Performance Indicator Reporting System, Electronic Asthma Quality of Life Questionnaire, Provider Asthma Assessment Form, Severe Asthma Algorithm, and Work-Related Asthma Screening Questionnaire (Long Version). The eTools presented during the workshops are detailed in Textbox 1. The eTools evaluated in this study were selected from tools developed by the ARU at Kingston Health Sciences Centre. A variety of types of eTools was selected to solicit feedback from participants on the benefits of and barriers to using different eTools in practice. Participants were asked a series of *yes* or *no* questions on whether they found the eTool to have value in primary care, whether the eTool was accessible, whether the eTool was user-friendly, and whether the eTool could support quality improvement. The feedback provided within the surveys was subsequently analyzed using descriptive statistics.

**Electronic tool (eTool) descriptions.**

- Asthma Action Plan Wizard
  An eTool that allows for automated generation of asthma action plans to provide patients with guidance on managing asthma
- Asthma Management and Outcomes Monitoring System
  An eTool that collects 69 data elements on asthma management and outcomes for quality improvement initiatives
- Electronic Asthma Performance Indicator Reporting System
  An eTool that collects guideline-based asthma performance outcome data at the point of care and generates reports supporting best practices and program evaluation
- Electronic Asthma Quality of Life Questionnaires
  A validated disease-specific quality-of-life questionnaire that enables inclusion of asthma quality-of-life data in electronic medical records and research databases
- Provider Asthma Assessment Form
  An evidence-based form to provide primary care providers with decision aids to support best practices regarding asthma assessment, diagnosis, and management
- Work-Related Asthma Screening Questionnaire (Long Version)
  A 14-item eTool designed to increase the recognition of work-related asthma in primary care

### Ethics Approval

The study was reviewed for ethical compliance by the Queen’s University Health Sciences and Affiliated Teaching Hospitals Research Ethics Board (TRAQ 6029444). Questionnaire responses were recorded anonymously. Study participants consented to the recording of the focus groups.

### Analysis

The qualitative data analysis began with the preparation and organization of the data. Video and audio recordings from the focus group discussions were transcribed verbatim by 3 members of the research team. Each team member’s transcription of the audio from the focus groups was reviewed and revised by all 3 research team members to ensure the accuracy of the transcription. Following transcription, a qualitative analysis was conducted to understand how best to integrate asthma tools and clinical guidelines into primary care EMRs.

The transcripts were analyzed using applied thematic analysis. Applied thematic analysis is an increasingly popular method to analyze qualitative data that captures patterns across raw data and structures the data into meaningful themes [17]. The research team focused the analysis on the central goal of learning how to integrate asthma tools and clinical guidelines into primary care EMRs. To determine specific themes and ideas, team members followed a predetermined step-by-step process. First, the transcript was read 3 times by each member of the research team to obtain a general sense of the data generated in the focus groups. Second, the text was read again to develop unique codes to represent the various ideas and comments raised by the focus group participants during the discussion. The coding process involved recording important quotations from the study that were related to the research question being analyzed. Each member of the research team maintained a memorandum during the data collection and analysis phases, making note of overarching themes and various connections within the data. The research team members met regularly to discuss and reach a consensus on the proposed codes. This process was repeated until no more unique codes were identified, at which point the research team concluded that data saturation had been achieved.

A combination of mind maps, tables, charts, and discussions was used to explore overarching themes pertaining to understanding how to integrate user preferences for asthma tools and clinical guidelines into primary care EMRs. Each member of the research team arrived at their own conclusions regarding the data themes. The themes proposed by the research team members were reviewed and re-evaluated over 4 team meetings. This process was repeated until consensus was reached on the major themes of the focus groups. Following this, the research team reviewed the transcripts and quotes to identify the best quotations to represent each of the key themes.

Throughout the data analysis, the members of the research team engaged in reflexivity. Reflexivity is an important aspect of qualitative research that consists of reflecting on the process of research and how an individual’s values and views may influence the findings of qualitative research [18]. Before completing the first step of the study protocol, research team members met to discuss how their backgrounds could influence the results of the study and the potential biases that may exist. By practicing reflexivity, research team members discovered that the shared experience in the ARU at Queen’s University and their knowledge of existing literature on eTools for asthma care could influence the data analysis. The team continued the reflexivity process throughout the data analysis process, with researchers recording additional potential biases and background knowledge in their analysis memoranda.

## Results

### Qualitative Analysis

#### Overview

Qualitative analysis of the focus groups revealed 7 key themes for integrating asthma tools and clinical guidelines into primary care EMRs across Canada (Textbox 2). The seven themes were (1) designing outcome-oriented tools, (2) gaining stakeholder trust, (3) facilitating open lines of communication, (4) prioritizing the end user, (5) striving for efficiency, (6) ensuring adaptability, and (7) developing within existing workflows.

**Key themes.**

- Outcome-oriented design
  Electronic tools (eTools) should be designed to achieve a specific outcome. Tools should include features to assist in reaching outcomes, including benchmarking, goal setting, trends, and incentives to create drive in the user.
- Prioritizing the end user
  When designing an eTool, the end user should be the most important consideration. This includes understanding the resources available to the user, incorporating provider preferences, and ensuring patient access.
- Developing for existing workflows
  For eTools to scale across the province and country, they must be incorporated into existing workflows through vertical integration and interoperability within the electronic medical record (EMR) environment.
- Gaining stakeholder trust
  Gaining the trust of various stakeholders involved in the process of care is crucial to facilitating uptake of a new eTool. This includes physicians, patients, nurses, clinic managers, and EMR vendors.
- Open communication
  Facilitating open lines of communication between provider and patient while also maintaining communication between developer and user is crucial for sustained eTool implementation.
- Ensuring adaptability
  The ability of an eTool to be flexible for the end user is important to ensure that the tool fits the needs of the user.
- Striving for efficiency
  Prioritizing the efficiency of the tool is critical for broad integration and incorporation into EMRs. Tools can improve efficiency and serve as a benefit or decrease efficiency and act as a barrier to integration.

#### Outcome-Oriented Design

A major theme emphasized across the focus groups was ensuring that the tool is designed to achieve specific, measurable outcomes. Focus group participants highlighted features such as the ability to view provincial and federal benchmarks, goal-setting features, visualization of trends, and performance incentives as ideal components of a newly designed eTool. Developing eTools with specific, quantifiable, and attainable goals for improving adherence to clinical guidelines was cited as an important component for demonstrating proof of value for any new tool. Providers highlighted the ability of an eTool to provide information on benchmarking to motivate them in their own practice:

Last piece I’ll say is just the benchmarking, I find it incredibly motivating you know, to be able to be shown how I’m doing against other providers and that’s another thing that sort of gets you, gets you moving.

Physicians also noted their desire to view their own practices in the context of the wider provincial average to evaluate how their outcomes aligned with their colleagues’ across the province:

...there is an under reporting or an under value of how many people in my practice actually have asthma listed as a diagnostic criterion, which means there’s probably an under performance in terms of managing asthma if I haven’t even identified some of the population that is having it, based on where we expect the province or the rates of asthma to be. So, I certainly see value in at least determining what’s my Delta, how far am I from a provincial average for what I see in my region.

Participants also discussed how focusing on a specific outcome and setting a goal to improve the outcome of interest through incremental steps can serve as a motivator:

I want to look at it you know what’s the outcome that we’re chasing on it, and I think many people if there’s small achievable targets, they’ll—you know something captures their attention they’ll do it.

Placing an emphasis on designing tools to meet specific outcomes is crucial for the integration of the technology into EMRs. Without the ability to evaluate specific outcomes, it is less likely that users will use the tool in their practices. As such, incorporating benchmarking and goal-setting functionality within an eTool can motivate a user and facilitate adherence to best-practice guidelines.

#### Prioritizing the End User

Focus group discussions emphasized the importance of the end user in developing a new tool for primary care EMRs. Understanding how the end user will use a new tool in the context of their own practice is important to ensure that new tools can be integrated into multiple types of practices. One of the most important considerations stated by focus group participants was ensuring that they had sufficient resources to effectively integrate a new tool into their practices. In defining resources, participants noted that the term “resources” can refer to human capital, mental energy, and time. The availability of resources to potential users is an important consideration for eTool developers, as stated by a physician:

There’s so much opportunity, but resources are limited so you have to be able to target it and focus it on the right places and the only way to do that is with the data to understand where those resources need to go.

Understanding the end user also means taking on the user’s perspective on why an individual would adopt a new tool. Through this process, incentives were found to be an important factor influencing a physician’s decision whether to adopt a new tool. Although there is an array of incentives, the discussion tended to focus on financial incentives. The desire for some form of incentive was very clear, with a participant remarking that “They truly will only do the things that we have funding for. Funding for in terms of payments for bonus or incentive...”

Prioritizing the end user also extends to patients. Both physicians and patient participants described how visualizations of data can be of benefit to better understand the data provided by the tool. Visualization of data provides a fast and clear format through which patients (and physicians) can see the effectiveness of a tool, as remarked by a participant:

I’ve had patients say it is really helpful to see how that tool is able to give me a visual on how this has improved my life. So certainly, that one is—is nice to be able to have, to show. And I think patients glean a lot from that.

Thus, prioritizing the end user in the design process is a central component of user satisfaction. Understanding the perspectives of the end users includes understanding their desires and needs. The happier the user is with a proposed eTool, the more likely it is that their behavior will be sustained.

#### Developing for Existing Workflows

Physicians in the focus groups reiterated the importance of developing new eTools to fit within their existing workflows. Participants felt that this feature was a requirement to scale an eTool over many practices and vendors. Participants noted that, if a new tool was proposed that fell outside a physician’s normal workflow, it would constitute a major barrier to facilitating uptake:

It has to do with integration into the EMR, so you don’t have to leave your environment, that just doesn’t work. So, the integration of these apps is really quite critical.

Despite the potential barriers and functional limitations that can arise in developing an eTool to fit within existing workflows, physicians also noted that, if the barriers to integrating eTools into an EMR are overcome, an embedded eTool can be of great benefit and improve patient care:

Clinician decision aids are really helpful, you don’t have to go outside your EMR to utilize them and that’s sort of key to make things as efficient as possible.

The most important feature identified to enable the adoption of a new tool into existing workflows was the “full” integration of the tool into the EMR currently used by the provider. Participants made it clear that having tools that function within their current EMR is essential as there is too much friction for tools that operate outside a physician’s normal EMR environment. Thus, targeting specific EMR vendors to reach the highest number of users is an important consideration.

#### Gaining Stakeholder Trust

Gaining the trust of stakeholders across the continuum of care was deemed crucial to integrating new technologies into primary care and was an important topic of discussion across the focus groups. From a physician perspective, one of the most common concerns was the accuracy of the data provided by the new tools, as stated by a participant:

If we’re using a dashboard or whatever surveillance system we’re using again, is it going to accurately be able to capture those patients who may not need things done in the cookie cutter fashion like maybe for some people that’s not appropriate and is there a way to capture that part of their care?

Understanding the potential value of a tool was also noted as being important to participants. However, proving the value of an eTool can often be a difficult task, as suggested by a patient participant who discussed needing to see the value when using a new tool:

My questions always are: what is this, who is this for, what is the benefit, and what is the value? As a patient, if I see the value that is the sign for you and me, because we are partners in my care, then perfect. It’s going to be easy for me to understand what we are talking about and what is the value. So, I need to be convinced.

The importance of gaining stakeholder trust when integrating a new eTool cannot be overstated. If providers and patients do not trust the accuracy of a new tool, they will not use it. Furthermore, for tools that have proven their accuracy, if users do not see the value in using the tool for themselves and their own practices, they will stop using the tool, threatening its longevity.

#### Open Communication

Open communication between partners in care was another important theme extracted from the focus groups. Participants noted how tools can facilitate improved communication between patient and provider:

It is near impossible to remember you know where, when you’re seeing a patient face-to-face you know, remember all those indicators but with tools, they can help, I think there’s value to using them.

The theme of open communication extended to the dialog between end users and tool developers. In marketing a new tool to patients and health care providers, users must be well informed of what the tool is and is not used for. A recurring fear that health care providers shared about adopting new eTools was the possibility of information being extracted from the tools to be used against them, with a participant stating the following:

I think on the other side there’s always this fear that the data is somehow going to be used for, you know, negotiations or if it’s in the wrong hands is going to be used against the physician in some sort of way which, which is obviously far from the truth.

The fear that physicians shared was stressed several times across the focus groups, with another participant saying the following:

One of the first things doctors ask me how, how will be, how will my information be used to punish me? And there is that suspicion always when we’re sharing data anywhere that it is going to go beyond us, whether it’s going to be sold, or worked with or managed, by governments or Pharma or you name it, there’s a fair bit of resistance there so that’s an issue.

Hence, it is crucial for developers to be transparent about the purpose of the tool and how it will be used by potential researchers. If physicians fear that the data collected by the tools will be used against them or to evaluate them negatively, it is unlikely that they will adopt the tool.

#### Ensuring Adaptability

Ensuring the ability to adapt eTools to the format that best suits the user was another prominent theme across the focus group discussions. The adaptability of the tool was a desired feature for both providers and patients. An example of the importance of adaptability was highlighted by a physician in one of the focus groups, who stated the following:

I expect that the tool will be the way that I read and then if it’s not formatted the way I read I want to be able to change it into the format that will make sense for me.

The discussion on adaptability developed into discourse on the optimal time at which to make use of an eTool, with a particular emphasis on when to administer the patient component of an eTool. Participants discussed various options for the best time to administer a patient questionnaire, such as during the visit with the patient in the room, while the patient is in the waiting room, or before the visit. The patient participant emphasized the benefits of completing questionnaires in the comfort of their own home before the visit, stating the following:

...something that I always say you know we should have those questionnaires um beforehand...I would prefer having those questionnaires [sent] electronically—or by mail if you are sending the PDF—so the person can fill out those questionnaires in the relaxed environment of home, when they can have the time to think and really make the reflective exercise of the information that they are providing.

An adaptable eTool can fit within a variety of primary care practices and serve a diverse cohort of patients. Thus, adaptability is an important feature to incorporate for developers who want their tools to scale across a broad spectrum of practices to suit the needs of large urban practices, small rural practices, and patients.

#### Striving for Efficiency

One of the central themes across both focus groups was the idea of efficiency. All participants emphasized the importance of time for the implementation of new tools. Health care providers discussed time as a scarce resource in their professional practices:

There’s always potentially you know an element of time or change in workflow or that sort of thing that we need to consider if we’re going to implement something, right? How much time will this take to complete?

The discussion regarding efficiency also included dialog on when the optimal time is to provide an eTool or the like. A physician in one of the focus groups also took on the perspective of the patient and stated the following:

I would like it in the waiting room, I mean, I would love to have something to fill out, because I mean there is nothing else to do it is the perfect opportunity. I think the way I would prefer it to happen would be to get an email a couple days before an appointment and have the opportunity to fill out but if I don’t, then I’m handed a tablet at the appointment visit to fill it out right, I think you have to use both strategies, not one or the other. It’s both.

The theme of efficiency also extends to efficiently presenting data to end users. Both health care providers and patients noted their preference for data to be recorded in a succinct format:

We have to make it as easy as possible; I think that’s kind of the key. Whatever data needs to be inputted, it’s got to be easy, otherwise people are not going to do it.

Participants made it clear during the focus groups that time was one of the most important considerations in adopting and using a new eTool. From data entry to tool use and visualization of data, efficiency is a crucial component to ensure that both providers and patients continue to use the tool over time.

### Quantitative Analysis

Focus group participants completed 2 surveys. A quantitative analysis was completed on all surveys provided to the focus group participants in conjunction with the findings of the qualitative analysis from the focus group discussions.

#### Asthma Indicator Survey

A total of 24 indicators were rated on a scale of 1 to 5 for their clarity, relevance, and feasibility, as well as an overall rating of 1 to 5 for their use as an indicator of asthma care. The indicators with the highest mean overall rating were smoking cessation support (mean 4.6, SD 0.79), frequency of emergency department visits (mean 4.5, SD 0.55), and monitoring of asthma using objective measures (mean 4.4, SD 0.54). The lowest-rated indicator was the assessment of reasons for poor control (mean 2.7, SD 1.38; Table 1).

The indicators that participants rated highest for clarity of measurement were tied between reliever use, hospitalizations for asthma, and inhaler technique (4.6). The indicator that respondents deemed most feasible to implement within an eTool was specialist care after ≥2 emergency department visits or hospitalizations for asthma. The indicators that participants rated as most relevant for use in primary care were tied between absenteeism from work or school and assessment of reasons for poor control. The results for clarity, relevance, and feasibility ratings for each indicator are shown in Figures S1-S3 in Multimedia Appendix 1. Participants preferred the PC-API indicators in 78% (7/9) of the cases where indicators overlapped between the PC-API and HQO indicator lists.

**Table 1:** Asthma indicator survey results—overall rating (average).

Asthma indicator	Overall rating, mean (SD)
Smoking support	4.6 (0.79)
ED^a^ visits	4.5 (0.55)
Monitoring using objective measures	4.4 (0.54)
Hospitalizations	4.4 (1.13)
Asthma control assessed	4.3 (0.52)
Asthma action plan	4.3 (0.95)
Primary care visits	4.1 (1.07)
Exacerbations	4.0 (1.15)
Diagnosis using objective measures	4.0 (0.58)
Anti-inflammatory therapy	4.0 (0.82)
Urgent care visits	3.9 (1.68)
Symptom-free days	3.9 (1.07)
Specialist referral	3.9 (1.07)
Follow-up with primary care or specialist after ED visit	3.9 (0.90)
Asthma-specific quality of life	3.7 (1.38)
Absenteeism from work or school	3.7 (1.25)
Routine care provider	3.7 (1.21)
Use of objective measures	3.6 (1.13)
Referred to or received asthma education	3.6 (1.62)
Actual asthma control assessed	3.6 (0.52)
Reliever use	3.5 (1.38)
Specialist care after ≥2 ED visits or hospitalizations for...	3.4 (1.40)
Inhaler technique	3.1 (1.07)
Assessment of reasons for poor control	2.7 (1.38)

^a^ED: emergency department.

#### eTool Survey

**Figure 1:**
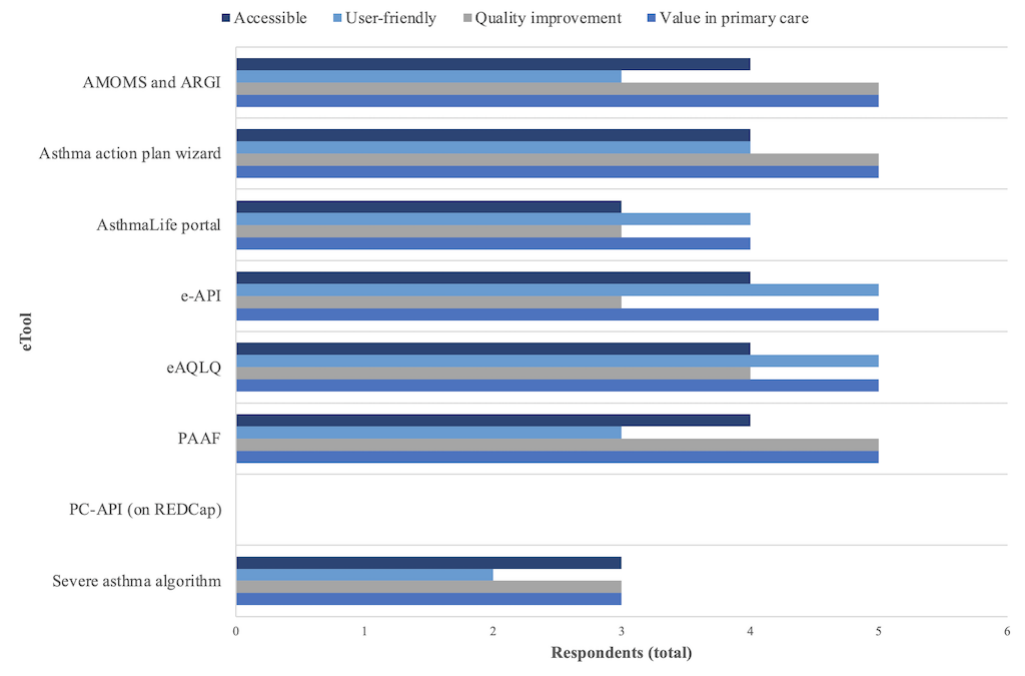
eTool survey results. AMOMS: Asthma Management and Outcomes Monitoring System; ARGI: Asthma Research Group, Inc; e-API: Electronic Asthma Performance Indicator Reporting System; eAQLQ: Electronic Asthma Quality of Life Questionnaire; PAAF: Provider Asthma Assessment Form; PC-API: Primary Care Asthma Performance Indicators; REDCap: Research Electronic Data Capture.

In total, 5 responses to the eTool survey were received. Of the 8 eTools presented, 5 (62%) were selected by all respondents as having value in primary care: asthma-specific data collection tools (Asthma Management and Outcomes Monitoring System and Asthma Research Group, Inc), the Provider Asthma Assessment Form, the Asthma Action Plan Wizard, the Electronic Asthma Quality of Life Questionnaire, and the Electronic Asthma Performance Indicator Reporting System. Participants’ ratings of the perceived benefits of the specific eTools demonstrated during the workshop are illustrated in Figure 1.

The eTool survey also elicited participants’ perspectives on which health care providers would benefit from the eTools demonstrated. Participants reported that certified asthma educators were most likely to benefit from the eTools (Figure S4 in Multimedia Appendix 1). The eTool survey also included an open-ended question on the potential barriers to the implementation of eTools. Respondents found the most common barrier to eTool implementation to be a lack of time to use the eTool (Figure S5 in Multimedia Appendix 1). In addition, 80% (4/5) of the respondents stated that they preferred to use eTools rather than paper or physical versions of tools.

## Discussion

### Principal Findings

This study used 2 focus group discussions to identify key considerations and barriers that exist when designing and implementing new primary care EMR eTools. The semistructured focus group sessions facilitated an inductive approach to addressing the research question. Analysis revealed that physicians, health professionals, and patients see the potential value and opportunity for eTools in EMRs to support adherence to best-practice clinical guidelines. However, there are barriers that must be overcome to successfully scale eTools across multiple practices and vendors. The 7 themes identified (outcome-oriented design, prioritizing end users, developing for existing workflows, gaining stakeholder trust, open communication, ensuring adaptability, and striving for efficiency) are important for developers to consider for successful integration of an eTool into primary care EMRs. Although studied within the context of asthma in primary care settings, these principles are likely relevant to other health conditions and care settings.

The 7 themes identified align with previous research on health information systems, such as the human, organization, and technology-fit (HOT-fit) model of health information system evaluations [19]. The HOT-fit model identifies 3 technological factors (system quality, information quality, and service quality), 2 human factors (system use and user satisfaction), and 2 organizational factors (structure and environment) that are important for evaluating a health information system. In the analysis of this study, each of the 7 themes identified aligned with one or several of the HOT-fit factors. System quality is related to outcome-oriented design and striving for efficiency. The HOT-fit service quality is directly related to the themes of gaining stakeholder trust and open communication. Regarding the HOT-fit human factors, system use can be addressed by developing for existing workflows and prioritizing the end user. Similarly, user satisfaction can be achieved by gaining stakeholder trust and striving for efficiency. Regarding the HOT-fit organizational factors, structure can be addressed by ensuring adaptability. Finally, the HOT-fit environment factor is related to prioritizing the end user and ensuring adaptability. Similarly, previous research on the recruitment of primary care physicians to an asthma KT study describes several barriers to primary care physician recruitment [20]. These include design-related challenges, the burden of time, and perceived threats to trust as some of the key barriers to primary care physician participation. Thus, the themes identified by the participants in this study align with and are supported by existing literature on health system evaluations.

Designing tools for specific outcomes was an important takeaway from the discussions. Practitioners may be more likely to use asthma tools that are directly related to the specific clinical outcomes they hope to improve and that are relevant to their practice. As a result, primary care clinical practice and adherence to best-practice guidelines may be improved. Without specific outcomes to target, users will be less likely to see value in the eTool, presenting a potential barrier to the scalability of the technology. Goal setting and benchmarking are recognized as some of the most effective forms of facilitating behavior change in primary care [21]. It is encouraging that participants demonstrated a desire for effective methods of behavior change to improve their own practices. As such, designing an eTool with specific outcomes is a crucial consideration for its success. Similarly, the adaptability of a proposed eTool is also important to consider as potential users come from a wide range of backgrounds and have varying degrees of comfort with eTools in general. As a result, the ability to adjust the features or components of an eTool to suit the practice of an individual user is an important consideration to facilitate widespread adoption.

Workflow efficiency emerged as a top priority for successful eTool integration and adoption. In short, if a tool saves time for the user, it will be used, and if it takes time from the user, it will not be used. Understanding the end user is an essential component of integrating an eTool into primary care EMRs and is crucial to facilitating uptake. When designing a new tool and presenting it to potential users, it is important to consider how the tool can be used in the process of care. Therefore, a workflow analysis before the launch or during the development of a new eTool is an important step in the design of new eTools. Understanding the resources required to use a new eTool is one of the most important design considerations. Many of the barriers faced when implementing new tools are related to the limited resources available to health care providers, which has been detailed in the literature [22]. Thus, for primary care physicians to incorporate a new eTool into their practices, the fewer human and financial resources required, the more likely it is that the eTool will be adopted [23]. This is especially true for implementing tools into primary care practices as the resources available to providers vary based on practice models and between jurisdictions. Similarly, our focus groups stated that time is one of the most important factors in deciding whether an eTool will be used. To save users time and increase the likelihood of adoption, eTools must be designed with efficiency in mind. The ability to record, view, and interpret data efficiently is a unique feature that can be provided by eTools. eTools are unique in their ability to offer evidence-based decision support in a timely manner directly to the provider at the point of care. In summary, if an eTool does not provide sufficient benefit to rationalize the time spent using it, it will not be used. The problem of time in the implementation of eTools has been a recurring theme in the literature on KT interventions in primary care [24].

Trust is another fundamental component of integrating asthma tools into primary care EMRs. For patients and health care providers to use an eTool, they must believe that it is accurate and will make a difference in the quality of care that they receive or provide. In the development of any new technology, gaining stakeholder trust is paramount for fostering uptake [20]. In the health care context, trust plays an even greater role in the adoption of new technologies owing to the sensitivity of the information and the consequences of errors in the information provided. Trust is facilitated through effective and open lines of communication. Effective means of communication between patients and providers have been highlighted in the literature as a fundamental component of KT for asthma in primary care [8]. Trust also extends to the relationship between the developer and end user. Across the focus groups, our participants reiterated their fear of data captured on eTools being used against them. As such, it is important that developers are able to effectively communicate the purpose of their tool and be fully transparent about the data being collected and how they will be used by researchers, governments, and affiliated organizations that can access the data. A method of communicating the purpose and safety of a new eTool can be supported by endorsements from credible individuals and organizations and through peer-reviewed publications.

An important finding that touches on several of the themes identified are the barriers that arise because of the hesitation of individuals throughout the continuum of care to adopt new tools. The reluctance of health care professionals and patients to adopt new tools is perhaps the most important barrier to overcome. Physician and patient reluctance is related to all the aforementioned barriers. The focus group discussions revealed that this human element is an important consideration in each theme, ranging from gaining trust in eTool adoption to prioritizing the efficiency of the eTool for patients. As a result, effective marketing of new tools and the involvement of stakeholders during development and implementation are vital for both keen and hesitant potential users to believe in the value of the tool and, ultimately, integrate it into their own practice or care [25].

Quantitative analysis of survey responses provided additional support to the statements made by focus group participants regarding the potential for eTools to improve adherence to best practices in primary care. The attitudes and beliefs of providers, particularly whether they trust the evidence upon which the eTool is based (ie, the quality of the guideline per se), may greatly influence eTool adoption. Participants demonstrated a preference for the PC-API indicators rather than the HQO indicators, providing additional support for PC-API indicators, which have been selected by expert consensus and proven to be feasible in primary care sites [26]. The responses of primary care physicians in our study provided additional evidence for the ability of PC-API indicators to be incorporated into an eTool. Furthermore, the eTool survey affirmed the view participants shared during the focus groups that eTools have the potential to improve adherence to best-practice guidelines in primary care. The positive reviews of eTools combined with physicians seeing value in most of the eTools presented during the focus group confirmed that primary care providers have a desire for eTools to be incorporated into their practices.

### Strengths

This study has important strengths that make it a unique contribution to the literature on understanding how to integrate asthma tools and clinical guidelines into primary care EMRs. First, our participants were experts in EMRs and eTools and knowledgeable on the eTools available to primary care providers. As a result, participants were familiar with eTools with successful and unsuccessful implementations and would have expertise on how to achieve a successful implementation. In addition, the use of mixed methods in this study was a major strength. The quantitative analysis provided numerical evidence to supplement our focus group findings, making our results more robust.

### Limitations

There are several limitations to this study. The COVID-19 pandemic affected the participation of OntarioMD Peer Leaders, and as such, the focus groups comprised a small sample of 7 and 5 participants. The study is also limited by the geographic representation of participants, who all practiced or lived in Ontario, limiting the generalizability beyond the province. Future research should include a larger sample size and representation from other provinces. In addition, 3 of the focus group participants were familiar with some of the eTools as current users of at least one eTool presented during the demonstrations, which may have introduced sampling bias to the results.

### Conclusions

Primary care physicians, allied health professionals, and patients consider that eTools for asthma care present a unique opportunity to improve adherence to best-practice guidelines in primary care and collect performance indicators. Some important considerations for integrating asthma tools and clinical guidelines into primary care EMRs across Ontario and Canada include an outcome-oriented design, prioritizing the end user, developing tools within existing workflows, gaining stakeholder trust, facilitating open communication, striving for efficiency, and ensuring adaptability. Potential barriers to eTool integration into EMRs include a lack of understanding of the resources available and the reluctance of providers to adopt new tools. Understanding and incorporating the findings of this study into the design of new eTools for asthma may facilitate their integration into primary care EMRs and support adherence to best-practice clinical guidelines.

## Appendix

Multimedia Appendix 1 Supplemental material.

## Abbreviations

ARU: Asthma Research Unit
EMR: electronic medical record
eTool: electronic tool
HOT-fit: human, organization, and technology-fit
HQO: Health Quality Ontario
KT: knowledge translation
PC-API: Primary Care Asthma Performance Indicators

## Data Availability

The data sets generated and analyzed during this study are available from the corresponding author upon reasonable request.
