# Supplementary material for: Integrating User Preferences for Asthma Tools and Clinical Guidelines Into Primary Care Electronic Medical Records: Mixed Methods Study
Source: JMIR Form Res. 2023 Feb 21;7:e42767. doi: 10.2196/42767 (PMC9993230; doi:10.2196/42767)
Supplement: Multimedia Appendix 1 [file formative_v7i1e42767_app1.docx]

**Supplemental Material**

**Table S1: Asthma Indicator Descriptions**

| **Indicator** | **PC-API Description** | **HQO Description** |
| --- | --- | --- |
| Absenteeism from Work or School | Number of days missed from school or work due to asthma in last 4 weeks | Average number of days missed from school or work due to asthma in the previous 4 weeks |
| Asthma Action Plan | Percentage of patients with asthma who have received a written asthma action plan | Percentage of children and adolescents / adults with asthma who have a written personalized asthma action plan and who have had their asthma action plan reviewed in the previous 12 months |
| Actual Asthma Control Assessed | Percentage of patients with well-controlled asthma in last 4 weeks | Average number of asthma symptom-free days in the previous 4 weeks among adults with asthma |
| Anti-inflammatory Therapy | N/A | Percentage of adults with asthma with one or more appropriate indications who are prescribed inhaled anti-inflammatory therapy |
| Assessment of Reasons for Poor Control | N/A | Percentage of children and adolescents / adults with uncontrolled asthma who have had all their reasons for poor control addressed |
| Asthma Control Assessed | Percentage of patients with asthma who had their asthma symptom control assessed in last 6 months | Percentage of adults with asthma who had a structured asthma assessment in the previous 6 months |
| Asthma Specific Quality of Life | Patient's assessment of their asthma-specific quality of life | N/A |
| Diagnosis by Objective Measures | Percentage of patients aged 6 years and over whose diagnosis of asthma was confirmed by spirometry, peak flow measurement or methacholine challenge test; | Percentage of adults/children with incident asthma whose diagnosis is confirmed with lung function testing |
| ED Visits | Number (or %) of emergency department visits for asthma in last 12 months | Percentage of adults with asthma who visited the emergency department for an asthma-specific reason in the previous 12 months |
| Exacerbations | Percentage of patients with asthma who had ≥ 1 asthma exacerbation in the last 12 months | N/A |
| Follow-up with Primary Care or Specialist Post ED Visit or Hospitalization | N/A | Percentage of children and adolescents / adults who have a follow-up assessment in primary care or in an asthma clinic within 7 days following an emergency department visit or hospitalization for an asthma exacerbation |
| Hospitalizations | N/A | Percentage of children and adolescents / adults with asthma who were hospitalized for an asthma-specific reason in the previous 12 months |
| Inhaler Technique | Percentage of patients with asthma who demonstrated their inhaler technique regularly | N/A |
| Monitoring by Objective Measures | Percentage of patients aged 6 years and over with asthma who received spirometry in last 12 months | Percentage of adults with asthma who completed a lung function test in the previous 12 months |
| Primary Care Visits | Number of primary care visits for asthma in last 12 months | N/A |
| Referred or Received Asthma Education | Percentage of patients with asthma who were referred to a certified asthma educator | Percentage of children and adolescents / adults with asthma who have ever received asthma self-management education from a trained health care professional |
| Reliever Use | Number of short-acting beta2-agonist doses (2 puffs) per week in last 4 weeks | Number (or %) of beta2-agonist free days in last 4 weeks |
| Routine Care Provider | Percentage of patients with asthma who have a routine care provider | N/A |
| Smoking Support | Percentage of patients with asthma who are smokers having received advice/support to stop smoking | N/A |
| Specialist Referral | N/A | Percentage of adults with severe asthma or one or more appropriate indications who are referred to specialized asthma care |
| Symptom-free Days | Number (or %) of asthma symptom-free days in last 4 weeks | N/A |
| Urgent Care Visits | Number of urgent care visits for asthma in last 12 months | N/A |

**Supplemental Results**

**Figure S1.** Asthma Indicator Survey Results – Clarity Rating (Average**)**

**Figure S2.** Asthma Indicator Survey Results – Feasibility Rating (Average)

**Figure S3.** Asthma Indicator Survey Results – Relevance Rating (Average)

**Figure S4.** eTool Survey Results – Beneficiaries (Total)

(AMOMS=Asthma Management and Outcomes Monitoring System, e-API= Electronic Asthma Performance Indicator Reporting System, eAQLQ=Electronic Asthma Quality of Life Questionnaires, PAAF=Provider Asthma Assessment Form, PC-API=Primary Care Electronic Asthma Indicator)

**Figure S5.** eTool Survey Results – Barriers (Total)

(AMOMS=Asthma Management and Outcomes Monitoring System, e-API= Electronic Asthma Performance Indicator Reporting System, eAQLQ=Electronic Asthma Quality of Life Questionnaires, PAAF=Provider Asthma Assessment Form, PC-API=Primary Care Electronic Asthma Indicator)
